# Evaluating differential nanoparticle accumulation and retention kinetics in a mouse model of traumatic brain injury via K^trans^ mapping with MRI

**DOI:** 10.1038/s41598-019-52622-7

**Published:** 2019-11-06

**Authors:** Hunter A. Miller, Alexander W. Magsam, Aria W. Tarudji, Svetlana Romanova, Laura Weber, Connor C. Gee, Gary L. Madsen, Tatiana K. Bronich, Forrest M. Kievit

**Affiliations:** 1 Department of Biological Systems Engineering, University of Nebraska, 200 LW Chase Hall, Lincoln, NE 68583 USA; 2 Department of Pharmaceutical Sciences, University of Nebraska Medical Center, Durham Research Center I, Room 1036, Omaha, NE 68189 USA; 3 ProTransit Nanotherapy, 16514L St., Omaha, NE 68135 USA

**Keywords:** Biomedical engineering, Imaging techniques and agents

## Abstract

Traumatic brain injury (TBI) is a leading cause of injury-related death worldwide, yet there are no approved neuroprotective therapies that improve neurological outcome post-injury. Transient opening of the blood-brain barrier following injury provides an opportunity for passive accumulation of intravenously administered nanoparticles through an enhanced permeation and retention-like effect. However, a thorough understanding of physicochemical properties that promote optimal uptake and retention kinetics in TBI is still needed. In this study, we present a robust method for magnetic resonance imaging of nanoparticle uptake and retention kinetics following intravenous injection in a controlled cortical impact mouse model of TBI. Three contrast-enhancing nanoparticles with different hydrodynamic sizes and relaxivity properties were compared. Accumulation and retention were monitored by modelling the permeability coefficient, K^trans^, for each nanoparticle within the reproducible mouse model. Quantification of K^trans^ for different nanoparticles allowed for non-invasive, multi-time point assessment of both accumulation and retention kinetics in the injured tissue. Using this method, we found that 80 nm poly(lactic-co-glycolic acid) nanoparticles had maximal K^trans^ in a TBI when injected 3 hours post-injury, showing significantly higher accumulation kinetics than the small molecule, Gd-DTPA. This robust method will enable optimization of administration time and nanoparticle physicochemical properties to achieve maximum delivery.

## Introduction

Traumatic brain injury (TBI) is a leading cause of death and disability, contributing to 30.5% of all injury-related deaths in the U.S. with 1.7 million new cases annually^1^. Immediate biological responses to TBI include inflammation, oxidative stress, blood-brain barrier (BBB) breakdown, and oedema, which often result in lifelong physical, cognitive, and psychosocial impairments that can worsen as individuals age^2,3^. Current TBI treatments focus on stabilizing the patient, relieving intracranial pressure, and optimizing cerebral perfusion. There are no neuroprotective therapies that have improved neurological outcome in a large, multi-centre Phase 3 clinical trial^4,5^. Part of the difficulty in developing effective therapies for TBI is that systemic administration does not allow for therapeutically significant drug levels to accumulate and be retained in the brain for effective target engagement^5–7^.

Because of the difficulty in reaching effective drug concentrations in the brain, post-injury BBB opening is of particular interest. Some groups have sought to improve outcomes by minimizing BBB opening following TBI, but the opening also provides an opportunity for systemically administered drugs or nanoparticles (NPs) to reach and accumulate in the damaged region^8–10^. The degree of BBB opening varies temporally post-injury, providing a temporary window of opportunity for increased uptake of a therapeutic into the injury^11^. Reports of the time of maximum uptake have varied depending upon the agent being used, suggesting different therapeutic agents optimally cross the disrupted BBB at different times post-injury^11,12^. Thus, it is essential to describe the time-dependent permeability to any therapeutic agent so that it can be administered at the opportune time.

NPs represent a highly controllable method of drug delivery, as they can be designed to regulate release characteristics, their size can be controlled to control pharmacokinetics and biodistribution, and surface conjugation of targeting agents allows for attachment to specific sites in the body^13,14^. For TBI, the size of NPs can be optimized to allow rapid accumulation in the damaged tissue, like a small molecule drug, while still being large enough to be retained in the tissue, not diffusing away as quickly as smaller molecules^15^. This results in a phenomenon similar to the enhanced permeability and retention (EPR) effect seen in tumors^16,17^. Indeed, NPs have gained significant interest in a number of preclinical studies targeting brain injury^10,18–20^. Surface modifications can further increase blood retention time and allow for real-time imaging during administration^21^.

The ability to track NP distribution kinetics will help accelerate the discovery of lead formulations and provide opportunity for tracking efficacy in the clinic. Through the incorporation of fluorophores and magnetic resonance imaging (MRI) contrast agents (CAs), NP accumulation can be detected and quantified. One recent NP study used fluorophores to corroborate the transient nature of TBI-related BBB dysfunction^22^ previously shown using Evans blue and horseradish peroxidase^11^. While accumulation of fluorescent NPs can be detected using confocal microscopy, the number of animals required for sufficient temporal resolution is high especially when correlating with therapeutic efficacy. In MRI however, sufficient temporal resolution can be achieved in each individual animal dependent upon the sampling rate. MRI can be used to collect a dynamic series of images after injection of contrast agent (CA) to determine CA concentration at each time point. MRI also offers the advantages of describing immediate uptake of NPs and allowing for multiple imaging sessions from the same animal to enable assessment of NP uptake, retention, and BBB permeability over an extended time period.

Multiple kinetic models have been created to describe the underlying physiology that governs the movement of substances from the blood into surrounding tissue^23^. The modified Toft’s model is a commonly utilized two-compartment model that describes transport between the plasma and interstitial space in a highly perfused tissue through three parameters, the fractional plasma volume *v*_*p*_, the fractional extravascular-extracellular volume *v*_*e*_, and the volume transfer coefficient K^trans^^24^. K^trans^ has previously been used to describe BBB permeability in humans and in rats^25–29^ with MRI as well as computed tomography (CT)^30^. K^trans^ is the main parameter of interest when seeking to describe BBB function as it quantifies the rate at which molecules in the plasma space are transferred to the extravascular-extracellular space and is calculated based on the relation between CA concentrations in tissue and plasma over time^31^. K^trans^ is not solely dependent upon the barrier being traversed, in this case BBB compromised by a TBI, but also depends on the CA crossing that barrier.

In this work we extend the use of the K^trans^ parameter to evaluate and compare the permeation of different NPs across a disrupted BBB rather than as a measure of the BBB status. Use of a repeatable controlled cortical impact (CCI) mouse model serves to minimize inter-animal injury variance, allowing for attribution of varying K^trans^ to differences between NP varieties. We utilize various CA-conjugated NPs to allow for MRI monitoring of accumulation and retention in a mouse brain following TBI to assess the robustness of the method to different NP types. We test the efficacy of K^trans^ as a method of measuring NP uptake kinetics at various time points following CCI. We also show how this method compares to other more common means of measuring NP uptake including the change in longitudinal relaxation rate (∆R_1_) and concentration maps. Briefly, R_1_ (inverse of T_1_) describes the rate at which net magnetization realigns with the magnetic field, B_0_, after application of the RF pulse, thus ∆R1 is the change in this rate caused by the presence of a contrast agent or NP. This method holds promise for a range of NPs to enable rapid evolution of their properties to promote desired accumulation and retention kinetics in target tissue.

## Results

### NP characterization

Dynamic contrast-enhanced MRI (DCE-MRI) can be used to measure NP accumulation in a tissue of interest (e.g., damaged brain tissue) and various physiological parameters of that tissue based on the kinetic model used to most accurately represent that tissue^32^. T_1_-signal can be directly used to detect accumulation in the brain, but a series of T_1_-weighted images can be further analysed to more fully quantify tissue properties once NP relaxivity has been quantified using MRI^33^. Quantification of R_1_ relaxivity (Table 1) shows the relaxivity of PLGA NPs as 4.78 (s^−1^mM^−1^) with respect to Gd concentration in NPs compared with 4.1 (s^−1^mM^−1^) for Gd-DTPA, though the R_2_/R_1_ value for PLGA NPs was higher than that of Gd-DTPA, 5.32 compared with 1.12. While they show similar R_1_ relaxivities, the differing R_2_/R_1_ values indicate they are not highly similar in their function as CAs. In contrast to the PLGA NPs, the Pro-NP^TM^ and Cj-1 NPs showed lower R_1_ relaxivities of 2.63 and 2.60 (s^−1^mM^−1^ CA) respectively, and much higher R_2_/R_1_ values of 42 and 12.3 respectively. These differences in relaxivities between the CAs allowed us to test how our imaging procedures are affected by different contrast enhancing properties.

**Table 1:** Nanoparticle properties.

Particle	Size (nm)	Contrast Agent concentration [mmol CA mg^−1^ NP^−1^]	Polydispersity Index (PDI)	R_1_ Relaxivity [s^−1^ mM^−1^] at 9.4 T	R_2_/R_1_	Zeta Potential [mV]	Injection Dose
Cj-1	80	5.1 × 10^−5^	0.24	2.60	12.3	+ 13.1	0.9 mg mouse^−1^
PLGA	89	2.29 × 10^−5^	0.41	4.78	5.32	−28.8	0.2 mg mouse^−1^
Pro-NP	214	1.635 × 10^−6^	0.036	2.63	42.0	−10.3	2 mg mouse^−1^
Gd-DTPA	0.83	1.07 mmol Gd mg^−1^ Gd-DTPA^−1^	N/A	4.1	1.12	N/A	0.05 µmoles mouse^−1^

NP hydrodynamic sizes were also varied with Cj-1 NPs being the smallest at 80 nm, PLGA NPs having a similar size of 89 nm, and Pro-NP NPs having a size of 214 nm as determined by DLS. Gd-DTPA acts as a nice surrogate for a small molecule drug with a size of approximately 0.83 nm^34^.

### Pharmacokinetics

Assessment of the pharmacokinetic parameters, which describe the behavior of NP in the blood, is a common preliminary method to gather some predictive information on the functionality of an agent^35^. MRI data from the imaging sequence included several slices containing the carotid arteries, allowing for blood concentration measurements from the injection of NP to the end of the imaging period. Pharmacokinetic quantification of NPs^36,37^ (Table 2) showed slower elimination of all NP types in comparison to Gd-DTPA. PLGA NPs showed longest elimination half-life (t_1/2,elim_) at 44 minutes, Cj-1 and Pro-NP were similar with respective t_1/2,elim_ of 29.7 and 27.7 minutes. As expected, Gd-DTPA showed the shortest t_1/2,elim_ of 15.7 minutes. On the contrary all distribution phase half-lives (t_1/2,dist_) were similar, varying between about 3 and 5 minutes among the different contrast agents. Mean area under concentration-time curve (AUC) values were calculated for the 60-minute imaging session for each particle type. Comparison of AUCs showed a large variation amongst NP types, there existed a roughly 40-fold difference between means of Cj-1 and Pro-NP. Note the difference in AUC units between Gd-DTPA and the three NPs.

**Table 2:** Nanoparticle pharmacokinetic properties.

Particle	Distribution Half-life (t_1/2,dist_)^51^	K_el,dist_ [min^−1^]	Elimination Half-life (t_1/2,elim_)^51^	K_el,elim_ [min^−1^]	Area under concentration-time curve (AUC)
Cj-1	4.74 ± 1.58	0.16 ± 0.055	29.69 ± 2.53	0.024 ± 0.002	152 ± 59 [min mg mL^−1^]
PLGA	3.12 ± 1.33	0.25 ± 0.068	44.02 ± 14.23	0.017 ± 0.005	329 ± 130 [min mg mL^−1^]
Pro-NP	3.64 ± 1.79	0.23 ± 0.11	27.74 ± 10.19	0.029 ± 0.012	6116 ± 3200 [min mg mL^−1^]
Gd-DTPA	4.01 ± 2.47	0.23 ± 0.11	15.70 ± 8.50	0.056 ± 0.023	6.6 ± 3.8 [min mM]

### ∆R_1_ and Concentration maps

**Figure 1:**
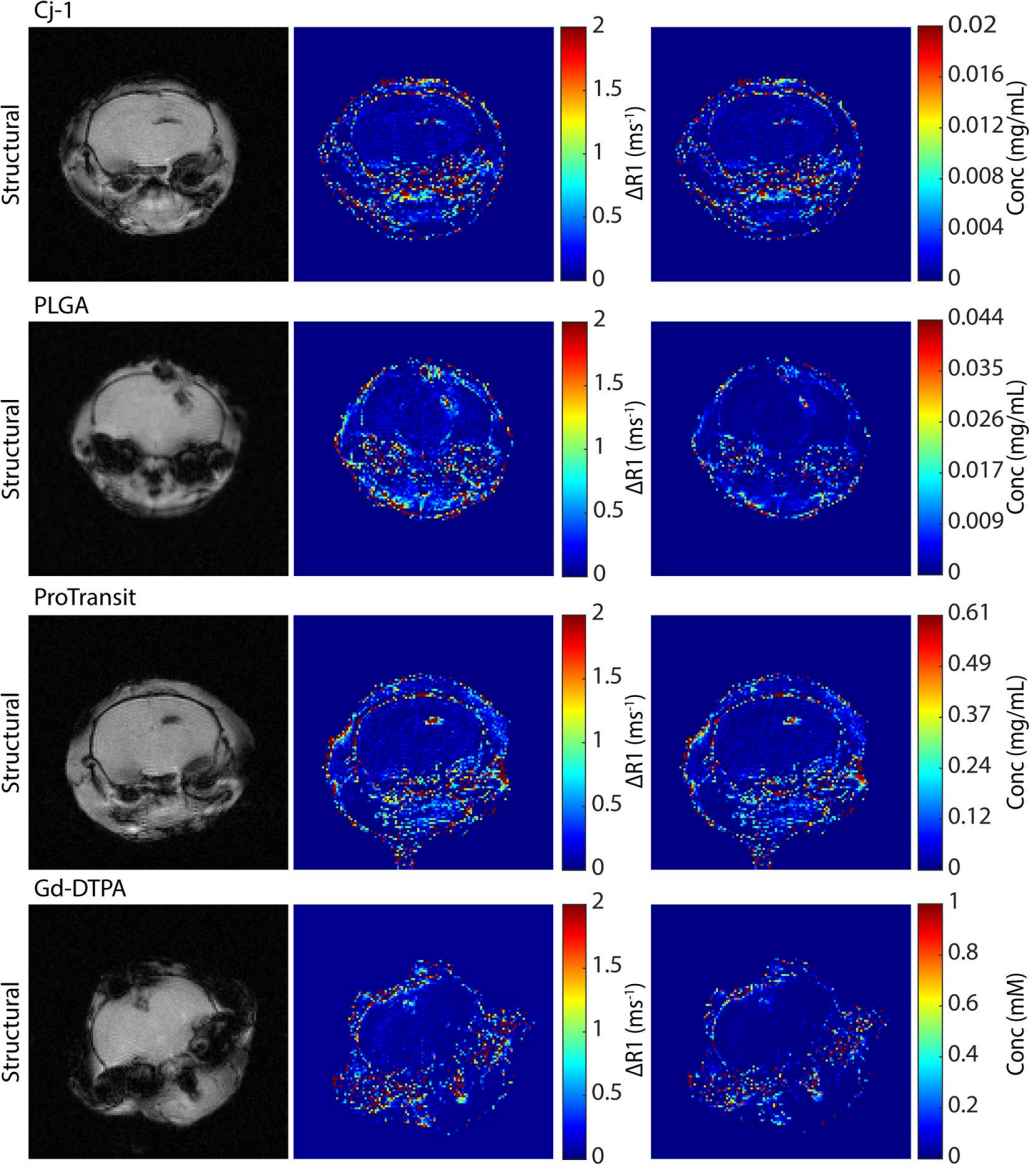
Structural MRI, ∆R_1_, and concentration maps for the three NPs and Gd-DTPA. Structural images show injury, dark area near top of brain, ∆R_1_ and concentration maps show change in R_1_ and NP concentration between final and initial scans. Highest ∆R_1_ and concentration values correspond to the injured regions in the structural images. Concentration units for Gd-DTPA have been displayed as mM and for NPs as mg/mL in an attempt to maintain use of units most familiar for these particles.

To evaluate BBB permeability based on NP properties following TBI, one of four NP types was injected in a CCI mouse model at a specific time point after injury. While an individual mouse was injected with only one NP type at one time point, different post-injury injection times and particle types were assigned to other individuals. T_1_-weighted MR images were collected over a 1-h period following NP injection, enabling visualization and quantification of change in R_1_ over time based on NP uptake. Variation between NP types in their contrast enhancement in a TBI can be seen in ∆R_1_ maps (Fig. 1), which compare R_1_ maps corresponding to specific time points with the pre-injection R_1_ map, but in all cases NPs and Gd-DTPA were observed in the damaged brain tissue further supporting the idea of an EPR-like effect in TBI. By dividing ∆R_1_ maps by the relaxivity values of the NPs, NP concentration maps were generated to provide a snapshot quantification of the amount of NP present in the TBI.

**Figure 2:**
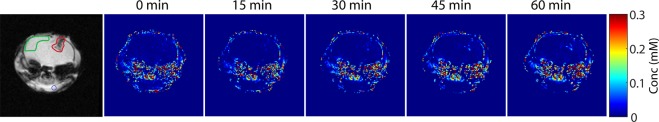
Region of interest model (left) used for calculating and analyzing average NP pharmacokinetics. Red corresponds to the focal injury, green to the contralateral region, and blue to the muscular reference region. Concentration maps (right) showing change in NP concentration over imaging session.

**Figure 3:**
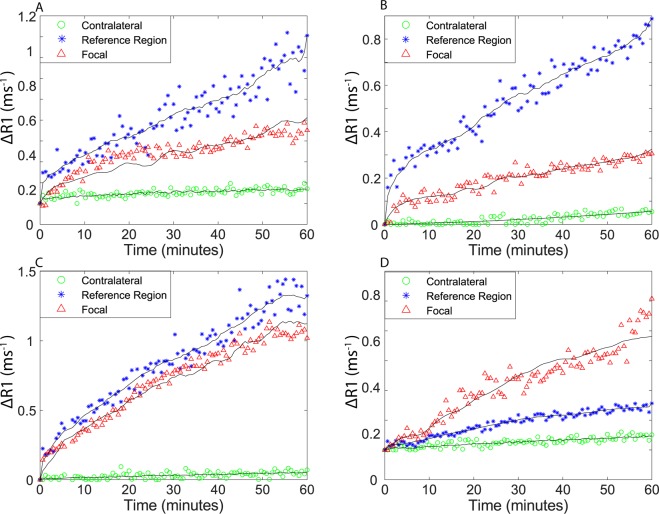
R_1_ values in TBI focal region, reference region, and contralateral over time calculated from DCE-MRI with (**A**) Cj-1 NPs (**B**) PLGA NPs (**C**) Pro-NP (**D**) Gd-DTPA. Sample regions are shown in corresponding colors in Fig. 2. Black lines show fitted curves for each region with each of the data points showing the experimental data.

NP accumulation was compared in the focal region of the injury, the contralateral region on the non-injured side of the brain, and a muscular reference region. ROIs were drawn in these brain regions (Fig. 2) to quantify NP uptake by location. NP concentrations in the brain were greater in the focal injury than the contralateral hemisphere across all particle types (Fig. 3), further supporting an EPR-like effect in TBI. These time-course measurements were then used to model (black lines in Fig. 3) NP uptake and accumulation kinetics in various brain regions to determine K^trans^.

**Figure 4:**
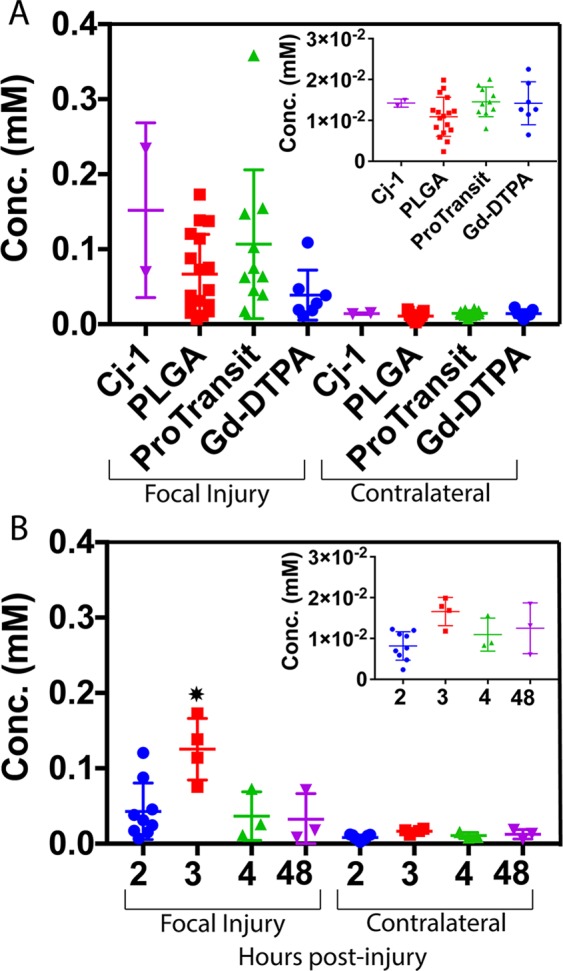
(**A**) Average final concentration values between NP types. No significant difference was detected between NPs despite differences in size and relaxivities. (**B**) Average final concentration values of PLGA NPs by post-injury injection time. *Indicates a significant difference (p < 0.01) determined using one-way ANOVA followed by Tukey’s post-test. Inlays show scaled concentration values in contralateral brain by NP type and injection time.

Final concentration values in the focal region were significantly higher (p < 0.01) than those in the contralateral hemisphere in PLGA NPs and Pro-NPs (Fig. 4A). Comparison of focal and contralateral final concentrations in Gd-DTPA showed a non-significant difference in means. No significant differences were seen between particles within the same brain region. When comparing PLGA NPs between injection times, significantly higher (p < 0.005) final concentration in the focal injury is seen following injection at 3 h post-injury compared to 2, 4, and 48 h time points (Fig. 4B). No difference was detected between injection times in the contralateral hemisphere.

### K^trans^ mapping

**Figure 5:**
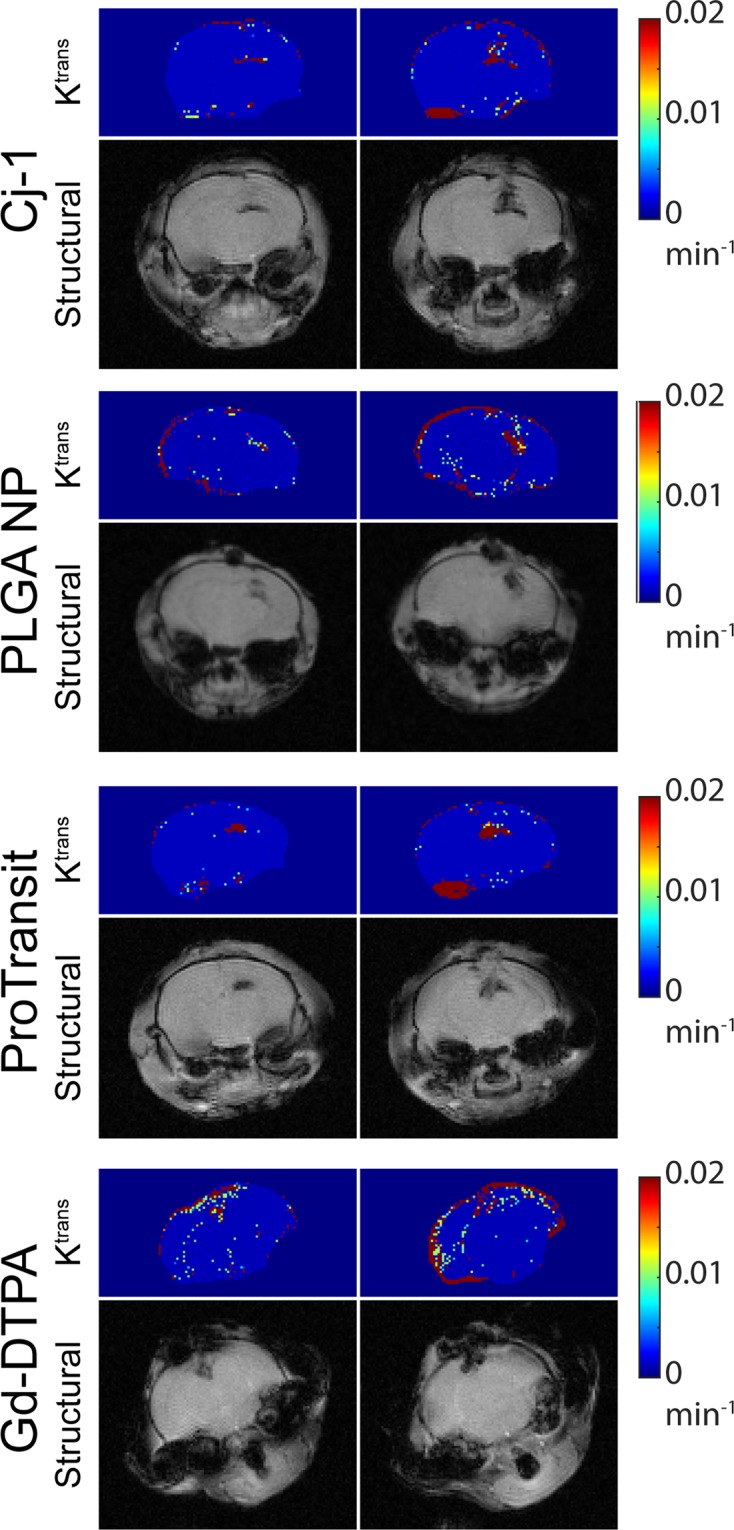
K^trans^ maps and corresponding structural MR images for all NP types. Structural images provide information on the injury location in the brain. Maximum values in K^trans^ maps (dark red) correspond to injury shown in the structural images (darker brain regions). This correspondence indicates areas of highest uptake are damaged tissue.

**Figure 6:**
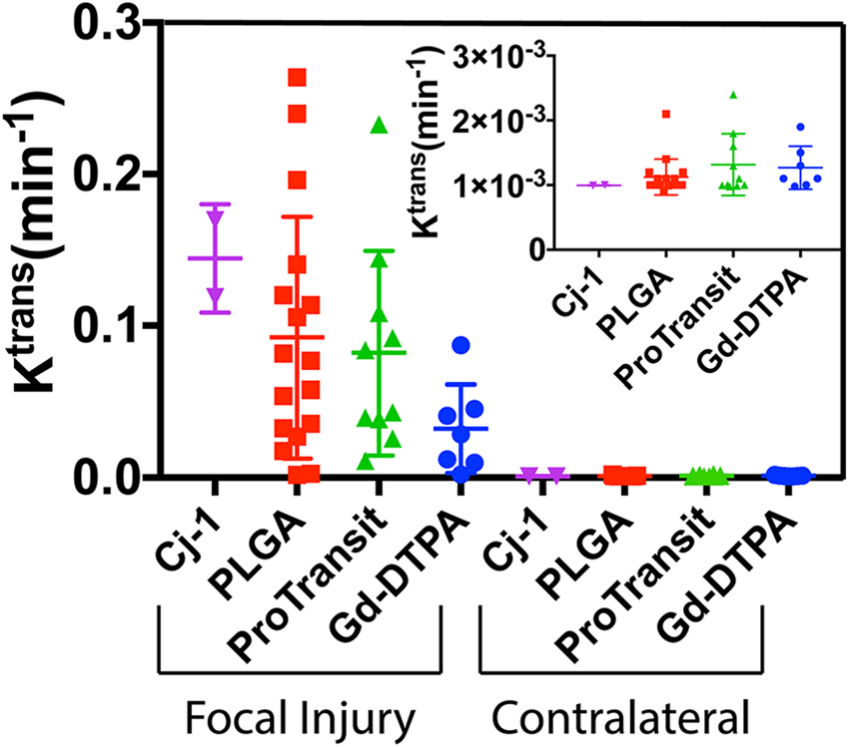
Average K^trans^ values between NP types. No significant difference was detected between NPs despite differences in size and relaxivity. PLGA NPs, Pro-NP and Gd-DTPA show a significant increase (p < 0.01) in K^trans^ in the focal injury as compared to contralateral brain. Inlay shows a scaled comparison between NPs on the contralateral side of the brain. Inlay shows scaled K^trans^ values in contralateral brain by NP type.

K^trans^ maps (Fig. 5) were generated using the integral form of the Kety equation and a highly vascularized muscular reference region to compare with the signal intensity changes in the tissue of interest^32^. Comparison of K^trans^ across NPs (Fig. 6) showed no significant difference between NPs within the same brain regions when averaged across all injection times. Significantly higher (p < 0.01) uptake was detected in the focal injury compared with the contralateral hemisphere for PLGA NPs and Pro-NP as well as the small molecule agent Gd-DTPA.

PLGA NPs were found to still accumulate in damaged brain when injected up to 48 hrs post-CCI though peak accumulation achieved when injected 3 h post-CCI. Focal K^trans^ values 3 h post-injury (mean of 0.170 min^−1^) were significantly higher than K^trans^ values for the same region at 2 (p < 0.01), 4 (p < 0.05), and 48 h (p < 0.01). This corresponds to a 125-fold increase in accumulation and retention kinetics over contralateral brain at the same 3 h post-injury time point (mean of 0.00135 min^−1^). Despite lower focal K^trans^ values, NP accumulation in focal injury when injected 48 h post-injury was still approximately 11-fold higher than contralateral brain.

**Figure 7:**
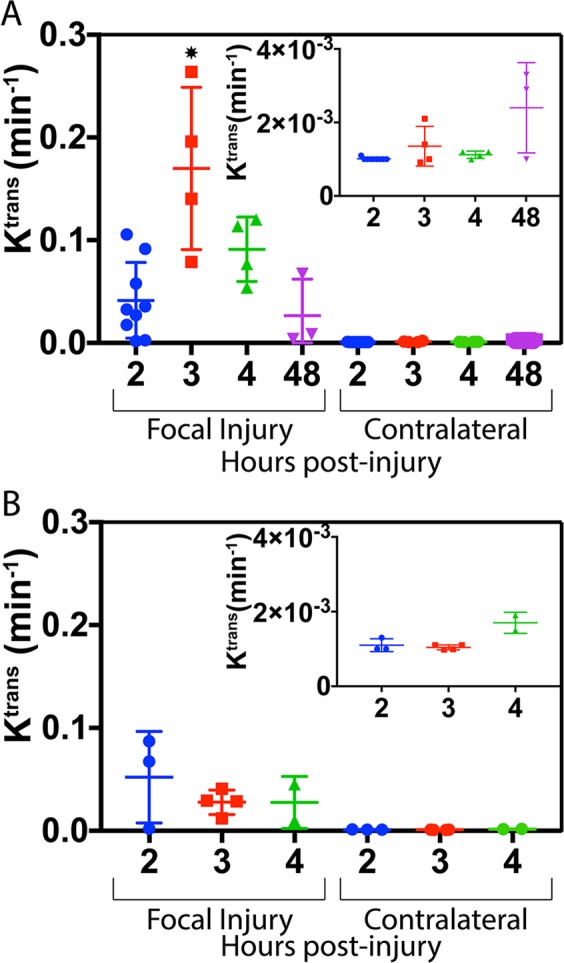
Average K^trans^ values for (**A**) PLGA NPs and (**B**) Gd-DTPA in the focal injury and contralateral brain when injected I.V. at various times after injury. NPs showed a time-dependent difference in uptake and retention kinetics as compared to Gd-DTPA where uptake and retention were not significantly affected by injection time. *indicates a statistical difference (p < 0.01) as determined by one-way ANOVA followed by Tukey’s post-test. Inlays show scaled K^trans^ values for each injection time on the contralateral side of the brain.

Comparison of K^trans^ values for PLGA NPs and Gd-DTPA grouped by post-injury injection time (Fig. 7) showed significantly higher (p < 0.05) accumulation and retention kinetics of PLGA than Gd-DTPA in the focal injury when administered 3 h post-injury. Despite showing much lower accumulation and retention kinetics in the focal injury than PLGA NPs when injected 3 h post-injury, Gd-DTPA K^trans^ in focal injury still showed an approximately 25-fold increase over contralateral brain indicating delivery to injured tissue. Gd-DTPA showed no significant difference in K^trans^ based on injection time. K^trans^ in focal injury (mean of 0.082 min^−1^) showed 62-fold increase over contralateral brain (mean of 0.0013 min^−1^) with Pro-NP. Cj-1 showed the a 144-fold increase in K^trans^ in the focal injury (mean of 0.1440 min^−1^) compared with contralateral brain (mean of 0.0010 min^−1^) though this difference was not statistically significant.

## Discussion

The lack of effective therapies for TBI represents a critical need for the development of new delivery strategies for improved target engagement. NPs are uniquely suited for delivery into the brain following a TBI because of an EPR-like effect caused by BBB breakdown^17^. However, there is still little information available on which NP properties ideally promote rapid accumulation and extended retention within the injury. Therefore, the goal of this work was to develop a robust method for assessing accumulation and retention of various types of NPs within a TBI using MRI that could be used to rapidly evolve desirable NP properties.

In this study we used three different NPs (negatively charged 89 nm PLGA NPs, negatively charged 214 nm Pro-NPs, positively charged 80 nm Cj-1 NPs) and the small molecule CA, Gd-DTPA. These different NPs were chosen because of their wide range of hydrodynamic sizes as well as different zeta potentials, which allows us to test the robustness of our imaging method to NP parameters. We first compared the relaxivities of the NPs to assess their ability to provide contrast in MRI. The similar relaxivity but differing R_2_/R_1_ values for PLGA NPs and Gd-DTPA indicate a similarity in their function as CAs, though not to a high degree. Pro-NPs and Cj-1 showed similarity to one another in terms of relaxivity but were lower than the PLGA NPs and Gd-DTPA. Despite the relaxivity differences, the imaging and processing protocols were shown viable with all NP types.

Despite the differences in R_1_ relaxivities between particle types, ∆R_1_ maps highlighted the injured region with higher values across all particles (Fig. 1). Quantification of different ROIs in concentration maps showed higher focal concentration with PLGA NPs administered 3 h post-injury compared with other administration times, similar to observations from Bharadwaj *et al*., using fluorescent NPs^22^. However, no differences were seen across administration times in Gd-DTPA, indicating a possible interaction effect on uptake between particle properties and post-injury administration time. This emphasizes the need for further analysis to optimize time of injection for unique particle types where a robust imaging method will allow for rapid optimizations.

The different NPs showed slight differences in pharmacokinetics following injection into TBI mice. The longer t_1/2,elim_ of the NPs likely corresponds to decreased renal clearance commonly observed for particles over ~8 nm in diameter as comapred to a small molecule like Gd-DTPA, which is cleared through the kidneys^18,19^. The increased plasma retention times of NPs may lead to increased uptake by elongating the time period during which transfer from plasma into the brain injury can occur. The unique pharmacokinetic parameters of Gd-DTPA are likely the cause differences seen in its uptake curves (Fig. 3). Gd-DTPA uptake differs from the three NPs in that highest uptake is seen in the focal region, whereas NPs show higher uptake in the reference region. Gd-DTPA more readily moves into the extracellular space throughout the body while the NPs remain within the vasculature for a longer period. As the NP concentration remains higher in the blood pool, they provide further enhancement in the highly vascularized reference region. AUC values differed greatly between particles, though it must be noted that the units used for NPs are different than those for Gd-DTPA as concentrations of these agents are not typically expressed in the same units. AUC variation is also attributable to the differences between injection concentrations of NPs (Table 1). PLGA NP concentration was selected to match the Gd concentration of Gd-DTPA dose, ProNP concentration was selected based on previous antioxidant studies using the particle, and Cj-1 concentration was selected in effort to provide similar contrast enhancement to Gd-DTPA and PLGA NP. As particles differ in their contrast-enhancing properties, the concentrations required for enhancement also differ, making comparison of AUCs between particle types difficult.

There was no significant difference in contralateral concentrations between administration times in PLGA NPs or Gd-DTPA. This lack of difference was likely a result of BBB integrity being maintained at early time points following brain impact in the undamaged tissue. While the generation of ∆R_1_ and concentration maps at multiple time points for each individual provides valuable data, a full understanding of uptake and retention must incorporate kinetic information. The information a concentration map provides at some time *t* does little to describe what occurred before that point or what will occur after. It is also difficult to distinguish what CA is still in the blood pool and will be rapidly removed or what has permeated into the tissue pool and will be retained. To better quantify NP permeability across the disrupted BBB, we must account for both accumulation and retention of NPs in the damaged tissue. K^trans^ incorporates plasma and tissue concentrations over the entire collection period to calculate a single value for each voxel. By comparing the relation of plasma and tissue concentration over time, K^trans^ provides permeation information unavailable in a single concentration map.

Determination of K^trans^ has previously relied on knowledge of the arterial input function (AIF), which describes NP concentration in blood plasma (*C*_p_) at time *t* after injection^32^. Estimating the AIF is typically done in one of three manners: (1) Blood sampling via arterial catheter concurrent with the imaging procedure allows for assessment of *C*_p_ via spectroscopic methods, but the small total blood volume of mice limits the number of samples that can be collected creating poor temporal resolution of the AIF. (2) A general AIF model can be created by measuring and averaging *C*_p_ over time from a number of sample individuals and then used to analyse the DCE-MRI images from individuals outside the sample cohort^38^. This model neglects the possible variation that may exist between sample groups and individuals within those groups and does not account for variabilities in injection volumes through the tail vein. (3) The third method is to estimate the AIF from the images in the data set by converting the blood signal intensity to *C*_*p*_. This method is contingent upon the presence of a large blood vessel in the field of view and is susceptible to inaccuracies caused by partial volume effects. The method is also hindered by the need for both high temporal and spatial resolution to accurately estimate AIF, as increasing temporal resolution negatively affects signal-to-noise ratio (SNR) and spatial resolution^32^. K^trans^ maps can be estimated without the AIF, using the integral form of the Kety equation and a highly vascularized muscular reference region to compare with the signal intensity changes in the tissue of interest^32,39^. By calculating the ratio of the tissue of interest K^trans^ to the reference region K^trans^ we can measure the relative difference in permeability between healthy brain tissue and damaged tissue without the need for an AIF. Therefore, for these studies we used the Kety equation and a muscular reference region^32,39^ to determine K^trans^ and generate K^trans^ maps.

K^trans^ mapping showed significantly higher uptake kinetics in the focal injury than in contralateral brain with PLGA NPs and Pro-NPs (p < 0.01). A significant difference (p < 0.05) between contralateral and focal regions was shown in Gd-DTPA using K^trans^ but was not seen when comparing final concentration values, showing the higher sensitivity of the K^trans^ method. Indeed, comparison of final concentration values in focal and contralateral regions in Cj-1, PLGA, Pro-NP, and Gd-DTPA showed fold differences of 10.7, 5.5, 7.3, and 5.8 respectively. K^trans^ comparison between focal and contralateral regions showed far greater fold differences of 145, 125, 62, and 25 respectively, showing increased sensitivity of K^trans^ for detecting CA accumulation and retention.

Comparison of K^trans^ between focal injury and contralateral brain showed higher fold differences in the three NPs than in Gd-DTPA, providing further support of an EPR-like effect in TBI; NPs show a greater uptake and retention as shown by higher K^trans^ in damaged tissue compared with healthy tissue than the small molecule Gd-DTPA whereas differences in concentration were not as dramatic. This also suggests the occurrence of passive targeting, supporting the use of NPs in TBI. Uptake is limited in healthy tissue and increased in the damaged tissue where a therapeutic effect is desired.

Contralateral K^trans^ values at 48 h were curiously higher than those observed at other time points for mice injected with PLGA NPs, though the difference was not statistically significant. This increase was not observed in the concentration mapping carried out at the 1 hr time point (Fig. 4B), supporting the increased sensitivity of K^trans^ in situations of minor BBB disruption. This increase in K^trans^ may correspond to a spread of minor BBB breakdown from the focal region to other parts of the brain that was not detectible with standard concentration mapping imaging techniques and merits further investigation. The focal and contralateral uptake at 48 h post-injury may also indicate a wider treatment window than previously described, meaning NP treatments could still be effective even when administered at extended time points after injury^40^.

A potential limitation of this study is the possibility of B_1_ artefact creating error within T_1_ measurements, ultimately affecting K^trans^ maps. As uptake quantification from the dynamic image series is based on differences from the precontrast T_1_ scan, accurate T_1_ measurement is essential. Transmit radio frequency (B_1_) field inhomogeneities can negatively affect the accuracy of T_1_ measurements and typically increase in severity with field strength^41^. To compensate for B_1_ inconsistencies, intensity-based postprocessing methods can be applied^42^, though compensation can also be carried out through B_1_ mapping prior to dynamic sequence collection. A common B_1_ compensation method is the double-angle method, which serves to determine the true flip angle at each voxel by comparing two images with flip angles α and 2α acquired with a long T_R_ to minimize T_1_ effect^43^.

Precontrast B_1_ mapping was not performed in this study, though large B_1_ artefact was not apparent in collected images (Supplementary Data Fig. 1) at different time points in imaging series. Intensity-based postprocessing B_1_ correction^42^ was performed on a subset of images for comparison with uncorrected originals. Normalized original MR images and concentration maps were compared with their B_1_ corrected equivalents (Supplementary Data Fig. 2) though differences were minor, mean focal concentration values in original and corrected maps showed no significant difference (p > 0.05) when comparing a randomly selected sample of 8 mice with a paired t-test. The lack of strong B_1_ artefact may be related to the narrow field of view (FOV), 20 × 20 × 1 mm^3^, utilized for imaging. The inhomogeneities present over a field of this size may be less impactful than those present in an FOV for human imaging, which can be larger in area by a factor of 100 or more depending on the tissue being investigated.

In conclusion, our results show the efficacy of K^trans^ as a robust method for measuring NP uptake and retention kinetics in TBI across various NP types and relaxivity properties. This method will allow researchers to rapidly evolve NP characteristics to improve delivery. Using K^trans^ as a measure of NP ability to accumulate and be retained in target tissue allowed us to determine that PLGA NPs had the highest accumulation and retention kinetics when injected 3 hours post-injury, though uptake was still detected when NPs were injected 48 hours post-injury. Additionally, we showed the methods described in this paper are viable for a variety of NPs with varying relaxivity properties, allowing for future uptake characterization of other CA-conjugated NPs. The adaptability of this method highlights the utility of image-guided NPs for assessing accumulation and retention kinetics to correlate with therapeutic efficacy. This will allow for a more rapid advancement of lead NP-based therapeutics and hopefully help overcome the current therapeutic stalemate for TBI.

## Materials and Methods

### Materials

All chemicals were purchased from Sigma-Aldrich unless noted otherwise.

### MRI and image processing

All images were acquired using a 9.4 T (400 MHz for protons) 89 mm vertical bore magnet (Varian, Walnut Creek, CA) with a 4 cm Millipede RF imaging probe with triple axis gradients (100 G/cm max). Specific imaging sequences and parameters that were used are provided in the relevant Methods sections. All acquired images were then processed in MATLAB 2017 (MathWorks, Inc., Natick, MA) as described in the relevant methods sections.

### Ethics statement

All work with live animals in this study were performed in accordance with the protocols approved by the Institutional Animal Care and Use Committee (IACUC) at the University of Nebraska-Lincoln.

### Controlled cortical impact mouse model for TBI

The CCI mouse model of TBI was chosen due to its high reproducibility and low mortality rates among other models of TBI, as well as the common factors held between human contusions and haemorrhaging from similar concussive wounds^44^. Female C57BL/6J mice were purchased from The Jackson Laboratory at an age of 6 weeks and acclimated for 1 week prior to use. Anaesthesia was induced via inhalation of 4% isoflurane gas then the mouse was secured in a stereotaxic frame (David Kopf Instruments, Tujunga, CA) and maintained at 1.5% isoflurane for the duration of the procedure. After induction of anaesthesia mice were injected subcutaneously with Buprenorphine SR (0.1 mg kg^−1^). Hair was removed from the incision site on top of the head using a depilatory agent (Nair), then the scalp was disinfected with a betadine scrub followed by isopropanol wipes. Lidocaine (0.05 mL at 5 mg mL^−1^) was applied topically to the scalp as an analgesic. A midline incision of roughly 1 cm was made over bregma and a 2 mm cranial window was made over the left frontoparietal lobe (2 mm left and 2 mm posterior from bregma) using a surgical drill. A controlled cortical impactor (Hatteras Instruments, Cary, NC) with a 2 mm tip attached to the stereotaxic frame was used to impact the brain normal to the dural surface at 4 m s^−1^ to a depth of 2.5 mm with a dwell time of 80 ms to generate a moderate injury severity^45^. Post-impact, the incision was closed with surgical adhesive and the mouse was monitored until regaining consciousness. Mice entered the imaging study at various time points (as reported in the figures) between 1–48 hrs post-surgery. Surgery was performed on a total of 40 animals in this study, though data were fully processed for only 36 animals. Two animals died shortly after contrast injection, likely due to introduction of air bubbles from the tail-vein catheter into the circulatory system of the mouse, and data acquisition was stopped. Data from 2 other animals were excluded due to motion during data acquisition.

### NP synthesis

PLGA NPs were composed of the following materials: poly(lactic-co-glycolic acid) (PLGA) (5–25 mg) for the core, 1, 2-Distearoyl-sn-glycero-3-phosphoethanolamine-Poly(ethylene glycol) (DSPE-PEG) (1 mg) to stabilize the surface, DSPE-N-diethylenetriaminepentaacetic acid (DSPE-DTPA-(Gd)) (1 mg) for MRI contrast, and DiD oil (25 μg) for fluorescence imaging. The NP materials were dissolved in an acetone:methanol (85:15) organic phase, while 2% sodium cholate was dissolved in ddH_2_O as the aqueous phase. A nanoprecipitation technique was used to form NPs, wherein organic phase (1 mL) was added dropwise to the aqueous phase (10 mL) under rapid stirring. Organic solvent was evaporated for one hour to harden the NPs.

The Cj-1 construct represents a Mn-porphyrin-polymer conjugate. Mn(III) meso-Tri(N-ethylpyridinium-2-yl)-mono(N-carboxy-methyl)porphyrin was synthesized from meso-*tetra*-(2-pyridyl)porphyrin via a four-step procedure. Preferential mono-alkylation was achieved with *tert*-butylbromoacetate at feeding ratio close to 1:1 followed by quaternization reaction with an excess of ethyl iodide^46^. *Tert*-butyl protecting groups were then removed in alkali conditions (KOH/THF). Metallation was completed by treating the resulting porphyrin with an aqueous MnCl_2_ solution at pH = 9.0 for 2 h and the reaction product was purified according to a previously reported procedure^46^. The structure of the final product was confirmand by ^1^H-NMR and inductively coupled plasma mass-spectroscopy (ICP-MS). Synthesized Mn-porphyrin was then conjugated to poly(ethylene glycol)-poly(L-γ-benzyl-glutamic acid) block copolymer (PEG-P(Bzl)Glu) via ethylene diamine linkers. Briefly, PEG-P(Bzl)Glu copolymer was synthesized as described previously^47^. The lengths of PEG and PEG-P(Bzl)Glu blocks were 114 and 40, respectively, as determined by ^1^H-NMR. PEG-P(Bzl)Glu (1 eq) was reacted with 100 eq of freshly distilled ethylenediamine in dry DMF at 45 °C for 24 h. The product was isolated by precipitation with cold diethyl ether and dried under vacuum. Mn-porphyrin was conjugated to prepared block-copolymer via EDC/NHS chemistry and purified by dialysis (MWCO 3,500) against water. Based on ICP-MS analysis, grafting degree was 8 porphyrin units per polymer chain. In aqueous solutions Mn-porphyrin-polymer conjugates formed small micelles (about 80 nm in diameter) with uniform particle size distribution (PDI = 0.24).

Pro-NP were prepared by ProTransit (Omaha, NE) using a water-oil-water emulsion and solvent evaporation method^48^. First, a 3% w/v poly(vinyl alcohol) (PVA) solution was prepared by adding the desired amount of PVA into distilled water under magnetic stirring. After stirring for 2 h at 600 rpm and 75 °C, the solution was allowed to stir at RT overnight. The PVA solution was then vacuum filtered through a 0.22 µm polyethersulfone membrane. For nanoparticle preparation, 85 mg of the PLGA polymer and 9 mg of (+)-dimethyl L-tartrate (DMT, used to stabilize the surface) was dissolved in 3 mL dichloromethane (DCM) and allowed to stir overnight. To this polymer solution was then added DiD oil (8 µL, 50 mg mL^−1^), bovine serum albumin (BSA, 250 µL, 120 mg mL^−1^), and DSPE-DTPA-(Gd) (162 µL, 50 mg mL^−1^ in DCM). The preparation was then emulsified using a staged microtip probe sonicator set at an energy output of 55 W for 2 min, 40% amplitude over an ice-bath to form the initial water-in-oil emulsion. This primary emulsion was added into 18 mL of the 3% w/v aqueous PVA solution and sonicated for 3 min, 40% amplitude over an ice-bath to form an oil-in-water emulsion. The resulting emulsion was stirred overnight at 600 rpm under a chemical hood to evaporate DCM and to form Pro-NP. Afterwards, the Pro-NP suspension was kept in a vacuum desiccator with magnetic stirring to ensure that all DCM had evaporated. The formed Pro-NP was recovered by centrifugation at 24,000 rcf using a 50.2 Ti rotor, for 30 min at 4 °C. Pro-NP were washed three times with distilled water to remove excess PVA. The final pellet was resuspended in 18 mL distilled water, frozen at −80 °C in a freezer, and then lyophilized for 48 h to obtain dry powder.

### Phantom tube preparation

A 0.25 mM (11.25 mg mL^−1^) solution of PLGA NPs was prepared and then diluted with PBS to concentrations of 0.25, 0.125, 0.0625, 0.03125, 0.0156, and 0.0078 mM. Each concentration in the same volume of PBS (400 μL) were transferred into seven separate 0.5 mL glass test tubes. The test tubes were arranged in order of decreasing concentration and inserted into a custom-made holder to secure the tubes in the centre of the magnet during imaging. Dilutions for Pro-NP,Cj-1 NPs, and Gd-DTPA were prepared in a similar manner with different maximum concentrations, of 0.65 mM (20 mg mL^−1^), 0.025 mM (0.5 mg mL^−1^), and 0.5 mM respectively. Pro-NP phantom tubes, for example, had concentrations of 0.65, 0.325, 0.163, 0.0813, 0.0406, and 0.0203 mM with a tube of pure PBS as a blank.

### NP relaxivity characterization

NP relaxivity was calculated from MR images acquired using the 9.4 T MRI system described above. After phantom preparation, the NPs were placed in the scanner and cross-sections of the phantoms were imaged. A fast spin echo imaging sequence was implemented using the following parameters: TE = 32.42 ms, 128 × 128 × 3 voxels, 25 × 25 × 3 mm^3^ FOV, and a seven TR values ranging from 200–2000 ms in increments of 300 ms. The saturation recovery method was used to calculate the relaxation time T_1_ of each voxel by fitting the MR signal to the following equation:

1 $$S={S}_{0}(1-{e}^{-\frac{TR}{{T}_{1}}})$$

where S is the MR signal for a given voxel, S_0_ is the signal of that voxel at saturation, and T_1_ is the longitudinal relaxation time of the given voxel. ROIs were drawn over each tube and average T_1_-values calculated. Relaxivity was calculated for each NP by plotting against NP concentration and finding the slope based on the following equation:

2 $${R}_{1}=r\ast C+b$$

where R_1_ = T_1_^−1^, C is the NP concentration, *b* is the intercept of the line, and r is the relaxivity of the NP in units *mM*^−1^*s*^−1^.

### Mouse preparation for imaging

Prior to imaging, mice were induced via inhalation of roughly 3% isoflurane gas and maintained using roughly 1.5% isoflurane. Mice were secured in a cylindrical animal holder to fix the head and body position of the animal during imaging. A pressure-based breathing monitor (SA Instruments, Stony Brook, NY) was secured over the back of the animal and isoflurane levels were adjusted to maintain a breathing rate between 40 and 70 breaths per minute. A tail-vein catheter with a 27-G needle was inserted into the tail and secured with surgical tape. The catheter tubing connected the needle to a 1 mL syringe at the base of the animal holder, which remained outside the bore of the magnet during imaging. Mice were injected with 100 µL PBS containing either 0.2 mM (9 mg mL^−1^) PLGA NP in PBS (100 μL), 0.1 mM (2 mg mL^−1^) Cj-1, 0.65 mM (20 mg mL^−1^) Pro-NP, or 0.5 mM Gd-DTPA via the catheter followed by a 100 µL bolus of PBS ensure all CA was expelled from the catheter and into the animal. Individual mice were injected with a single dose of one NP type, the number of individuals in each NP group were: Cj-1 n = 2, PLGA n = 17, Pro-NP n = 10, and Gd-DTPA n = 7. Group sizes for Cj-1 and Pro-NP were selected based on NP availability. PLGA and Gd-DTPA group sizes were selected to investigate the effect of post-injury injection time on uptake both within and between groups.

### MRI data collection

Animal imaging was performed using the 9.4 T MRI system described above. Animals were imaged using a variable flip angle gradient echo sequence with two flip angles for rapid calculation of R_1_^49^. Animal imaging was performed on the same imaging system utilized for relaxivity characterization. The parameters for the gradient echo sequence were as follows: TR = 54.24 ms, TE = 2.73 ms, 128 × 128 data matrix, 10 slices each with a FOV of 20 × 20 × 1 mm^3^, and 5 averages for a scan time (temporal resolution) of 34 s. Two baseline scans were acquired prior to injection of the NPs using flip angles of 10° and 30° respectively. All post-injection scans used a flip angle of 30°. At the conclusion of pre-injection scans, NPs were injected through the tail-vein catheter immediately followed by acquisition of roughly 100 scans over the course of 1 h post-injection.

### Concentration mapping

Images were processed using in-house software in Matlab 2017. *R*_1_ was calculated using the variable flip angle equation in slope-intercept form:

3 $$\frac{S}{\sin (\alpha )}={E}_{1}\frac{S}{\tan (\alpha )}+{M}_{0}(1-{E}_{1})$$

where *α* is the flip angle, *S* is MR signal *E*_1_ = *e*^−TR*R1^, and *M*_0_ is a proportionality constant. By plotting the ratio of *S* to *sin(α)* versus the ratio of *S* to *tan(α)* for multiple values of *α* a curve is generated whose slope is equal to *E*_1_, from which *R*_1_ can be calculated. Concentration of the NP at a given time point was then calculated using the following equation:

4 $$C(t)=\frac{{R}_{1}(t)-{R}_{1}({t}_{0})}{r}$$

where *r* is the relaxivity of the specific NP injected into the animal, *R*_1_*(t)* is the R_1_ value at time *t*, and *R*_1_*(t*_0_) is the pre-contrast R_1_ value. NP concentration was calculated at every voxel to create concentration maps for each animal at every time point.

### K^trans^ mapping

Pharmacokinetic parameter K^trans^ was extracted from the concentration maps using the reference region equation, which is derived from the extended Tofts’ model:^50^

5 $$\begin{array}{ccc}{C}_{TOI}(T) & = & \frac{{K}^{trans,TOI}}{{K}^{trans,RR}}\ast {C}_{RR}(T)+\frac{{K}^{trans,TOI}}{{K}^{trans,RR}}[(\frac{{K}^{trans,RR}}{{v}_{e},RR})-(\frac{{K}^{trans,ROI}}{{v}_{e,TOI}})]\\  &  & \ast {\int }_{0}^{T}{C}_{RR}(t){e}^{(\frac{-{K}^{trans,TOI}}{{v}_{e},TOI})(T-t)}dt\end{array}$$

where C_TOI_(T) and C_RR_(T) are the concentration of NP at a given time in the tissue of interest (TOI) and reference region (RR), respectively. Muscle tissue was used as the reference region in all animals, which is shown by the blue ROI in the structural image of Fig. 2. A least squares curve fitting routine was employed using Matlab to extract K^trans^ for each voxel in the brain. Mean K^trans^ values from focal and contralateral ROI’s, shown as red and green ROIs respectively in Fig. 2, were recorded for each mouse, separated by NP type, and reported in Fig. 6. Mean K^trans^ values for PLGA NPs and Gd-DTPA were further grouped by post-injury injection time and are reported in Fig. 7.

### Additional pharmacokinetic characterization

NP pharmacokinetic parameters (Table 2) were calculated based on AIFs collected from ROIs drawn in carotid artery in posterior-most slices of MR images. ROIs were selected in either carotid artery based on noise and level of signal enhancement, so that AIF reflected expected maximum concentration values. AIFs were based on average concentration versus time curves from multiple slices of the same individual where possible. Averaged AIFs were fit to a biexponential using a nonlinear least squares algorithm in Matlab:^36^

6 $${C}_{p}(t)=A{e}^{-\alpha t}+B{e}^{-\beta t}$$

where *C*_*p*_ is the plasma concentration, *A* and *α* are the linear component and rate constant respectively corresponding to the distribution phase, and *B* and *β* are the linear component and rate constant respectively corresponding to the elimination phase. t_1/2,dist_ was calculated as the time between a point, A_1_, on a line with slope A and another point, A_2_, such that A_1_ is 2*A_2_. t_1/2,elim_ was calculated in the same manner instead using a line with slope B. Elimination constants, K_el_, were calculated as:

7 $${K}_{el,i}=\,\mathrm{ln}(2)/{t}_{1/2,i}$$

where *i* indicates the phase, distribution or elimination.

### Statistical analysis

All statistical analyses were performed using GraphPad Prism 7.0e. Average concentration and Ktrans values were compared between NP types or injection times using one-way ANOVA followed by Tukey’s post-test to determine where the statistical difference occurred between groups. Statistical significance was defined as any p < 0.05.

## Supplementary information

Supplementary information

## Data Availability

Research data will be shared in accordance with the norms of the research community and will be made available upon request.
