# Supplementary material for: Evaluating differential nanoparticle accumulation and retention kinetics in a mouse model of traumatic brain injury via Ktrans mapping with MRI
Source: Sci Rep. 2019 Nov 6;9:16099. doi: 10.1038/s41598-019-52622-7 (PMC6834577; doi:10.1038/s41598-019-52622-7)
Supplement: Supplementary file 1 — Supplementary information [file 41598_2019_52622_MOESM1_ESM.pdf]

Evaluating differential nanoparticle accumulation and retention kinetics in a mouse model of traumatic brain injury via K^{trans} mapping with MRI

Hunter A. Miller, Alexander W. Magsam, Aria W. Tarudji, Dr. Svetlana Romanova, Dr. Laura Weber, Connor C. Gee, Dr. Gary L. Madsen, Prof. Tatiana K. Bronich, Dr. Forrest M. Kievit*

Supplementary Material

Supplementary Methods

B_1 Correction

A sample of 8 animals were randomly selected and an image containing the TBI selected for each animal. Images were treated with a Matlab algorithm based on the procedure described by Cohen, DuBois, and Zeineh.[1] Briefly, the background noise level was determined using a smoothed histogram of pixel intensities and selection of the lowest 15% of signal intensities. Average signal intensity was calculated from non-noise pixels in thresholding and this value was used to fill all noise locations. The resulting image was then smoothed via convolution with a gaussian kernel. The original raw image was divided by the smooth image and the quotient multiplied by the average pixel intensity. Resulting corrected images were normalized to have an intensity range of 0 to 1 and used to calculate concentration maps for the imaging series. Uncorrected MR images were normalized in the same manner and used to calculate concentration maps for comparison with the corrected maps.

Supplementary Results

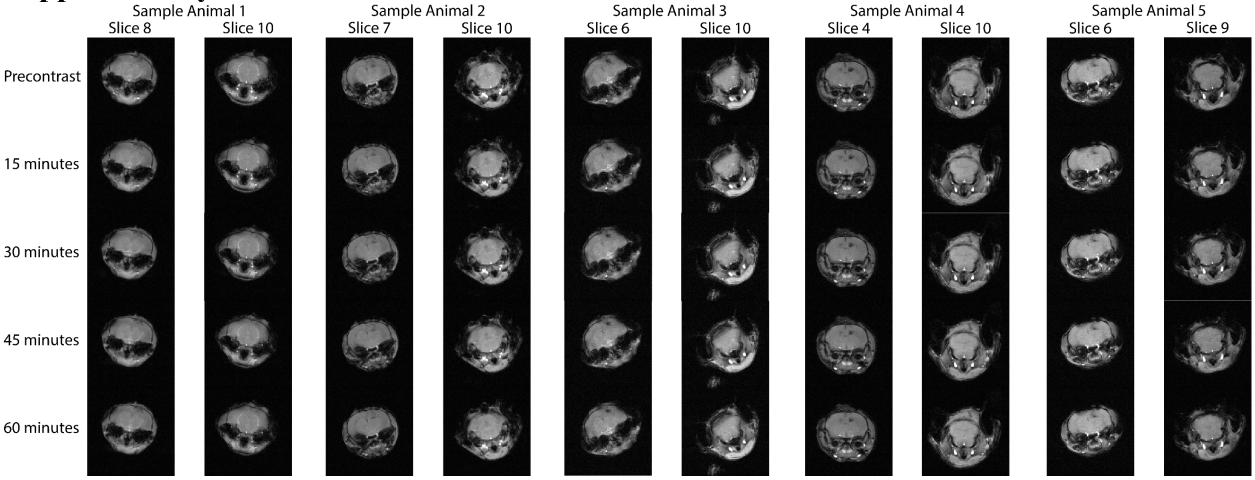

Supplementary Figure 1. Selection of sample animals for assessment of B_1 artifact present in imaging. In each animal a slice displaying the TBI is presented as well as a slice displaying the carotid arteries used in generation of pharmacokinetic parameters. B_1 correction was not performed on all images as inhomogeneity was deemed insufficient to merit it.

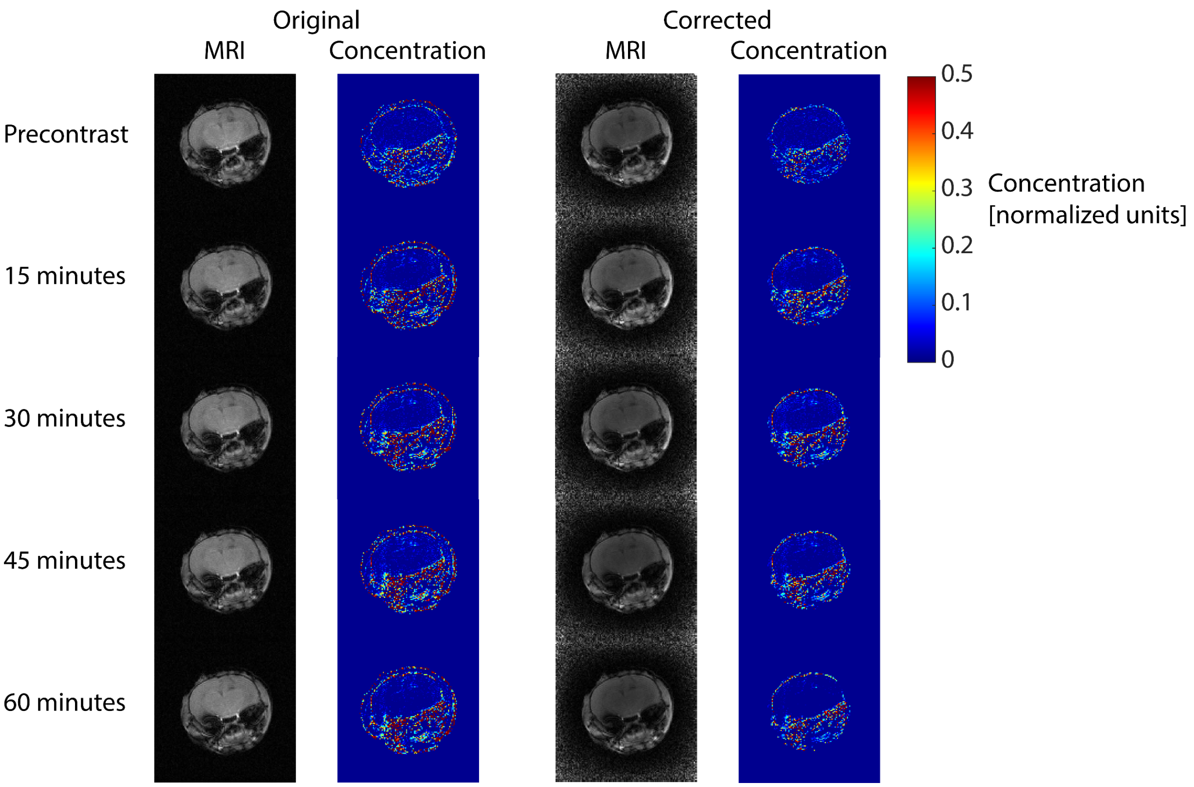

Supplementary Figure 2. Comparison of original MRI scans and concentration maps with the same scans after postprocessing B_1 correction and concentration maps from those corrected images. MRI scans were normalized so intensities ranged from 0 to 1 then used for concentration mapping. All concentration maps are on a scale from 0 to 0.5 as shown in the colorbar.

Supplementary references

1 Cohen, M. S., DuBois, R. M. & Zeineh, M. M. Rapid and effective correction of RF inhomogeneity for high field magnetic resonance imaging. *Hum Brain Mapp* **10**, 204-211 (2000).
